# Identification of pathogens from native urine samples by MALDI-TOF/TOF tandem mass spectrometry

**DOI:** 10.1186/s12014-020-09289-4

**Published:** 2020-06-23

**Authors:** Damir Oros, Marina Ceprnja, Jurica Zucko, Mario Cindric, Amela Hozic, Jasenka Skrlin, Karmela Barisic, Ena Melvan, Ksenija Uroic, Blazenka Kos, Antonio Starcevic

**Affiliations:** 1 Department of Biochemical Engineering, Faculty of Food Technology and Biotechnology, Zagreb University, 10000 Zagreb, Croatia; 2 Biochemical Laboratory, Special Hospital Agram, Polyclinic Zagreb, 10000 Zagreb, Croatia; 3 Division of Molecular Medicine, Ruder Boskovic Institute, Zagreb, Croatia; 4 Department for Clinical Microbiology and Hospital Infection, University Hospital Dubrava, 10000 Zagreb, Croatia; 5 Faculty of Pharmacy and Biochemistry, Zagreb University, Zagreb, Croatia; 6 Department of Biological Science, Faculty of Science, Macquarie University, Sydney, Australia

**Keywords:** Urine, Sample preparation, Pathogen identification, Proteomics, MALDI-TOF/TOF, 16S rRNA sequencing

## Abstract

**Background:**

Reliable high-throughput microbial pathogen identification in human urine samples is crucial for patients with cystitis symptoms. Currently employed methods are time-consuming and could lead to unnecessary or inadequate antibiotic treatment. Purpose of this study was to assess the potential of mass spectrometry for uropathogen identification from a native urine sample.

**Methods:**

In total, 16 urine samples having more than 10^5^ CFU/mL were collected from clinical outpatients. These samples were analysed using standard urine culture methods, followed by 16S rRNA gene sequencing serving as control and here described culture-independent MALDI-TOF/TOF MS method being tested.

**Results:**

Here we present advantages and disadvantages of bottom-up proteomics, using MALDI-TOF/TOF tandem mass spectrometry, for culture-independent identification of uropathogens (e.g. directly from urine samples). The direct approach provided reliable identification of bacteria at the genus level in monobacterial samples. Taxonomic identifications obtained by proteomics were compared both to standard urine culture test used in clinics and genomic test based on 16S rRNA sequencing.

**Conclusions:**

Our findings indicate that mass spectrometry has great potential as a reliable high-throughput tool for microbial pathogen identification in human urine samples. In this case, the MALDI-TOF/TOF, was used as an analytical tool for the determination of bacteria in urine samples, and the results obtained emphasize high importance of storage conditions and sample preparation method impacting reliability of MS2 data analysis. The proposed method is simple enough to be utilized in existing clinical settings and is highly suitable for suspected single organism infectious etiologies. Further research is required in order to identify pathogens in polymicrobial urine samples.

## Background

Urinary tract infections (UTIs) are the most common form of bacterial infections both in the general population and in hospital patients, attributing to nearly 25% of all infections [1]. UTIs are much more common in females than males. It is estimated that 40–50% of women will develop a UTI during their lives, and approximately 33% of women will have recurrent acute uncomplicated UTI [2]. Common primary bacterial uropathogens are *Escherichia coli, Staphylococcus saprophyticus, Enterococcus* spp.*, Proteus mirabilis,* and *Klebsiella pneumoniae*. While most common secondary uropathogens are *Staphylococcus aureus, Klebsiella oxytoca, Pseudomonas aeruginosa, Streptococcus agalactiae* and fungal pathogen *Candida* spp. [3–6]. Approximately 60–80% of all uncomplicated bacterial UTIs are caused by *E. coli*. Researchers have recognized that urine is not sterile and confirmed the importance of resident bacterial flora (urinary microbiota) in the lower urinary tract. Resident urinary microbiota is mostly composed of *Lactobacillus gasseri, Corynebacterium coyleae, Actinobaculum schaalii, Aerococcus urinae, Gardnerella vaginalis, Streptococcus anginosus, Streptococcus epidermis, Actinomyces neuii and Bifidobacterium* spp. [7, 8].

In order to identify microorganisms in clinical microbiology laboratories, most used methods are microbiological techniques which are still based on cultivation on different culture media [9]. Despite advances in genomics and proteomics, urine culture method is still the golden standard for the diagnosis of UTIs. Urine samples containing more than 10^5^ CFU/mL of a single microbial species usually indicate clinical relevance. However, there are significant shortcomings to these cultivation-oriented methods. The first limitation is the time required for the cultivation of microorganisms and subsequent identification [10]. Standard incubation times range from 12 to 24 h in order to enable reliable detection of the presence of uropathogens [11]. The second limitation is the requirement for fresh urine samples. Some of these limitations may result in overall negative urine cultures in up to 80% of cases, in many microbiology laboratories [12]. Unfortunately, a wide variety of sampling methods and inappropriate specimen transport are major cause of pre-analytical errors [13].

Various methods have been used for detection of microorganisms in clinical microbiology [14–16]. For fast screening of urine samples, flow cytometry (such as Sysmex analyser) has been used. However, urine flow cytometer is not able to provide bacteria identification [17, 18]. Genomic methods relying on DNA analysis, such as SeptiFast, FilmArray or GeneXpert, are being used, however they are still not approved by the FDA for UTI identification [14]. Usage of real-time PCR methods in the identification of uropathogens has been proven as feasible [19], however it is limited in its scope. Techniques using DNA sequencing regularly show more sensitivity compared to standard urine culture test. For this reason, bacterial identification relying on sequencing of the 16S rRNA genes is becoming a method of choice for detection of uropathogens in urine samples [20, 21].

Field of proteomics also offers methods for microbial identification, mass spectrometry (MS) being the most prominent one. MS platforms used include matrix-assisted laser desorption/ionization time-of-flight mass spectrometry (MALDI-TOF-MS) based analysis producing characteristic spectrum called peptide mass fingerprint (PMF), or less frequently used liquid chromatography tandem mass spectrometry (LC-MS/MS) based peptide sequencing. LC-MS/MS depends on initial isolation of bacterial colonies from urine and their subsequent cultivation [22, 23], while MS based analysers claim ability to directly process samples or swabs. Today, MS-based analysers are in routine use, such as the Bruker BioTyper (Bruker Daltonics) and VITEK MS Plus (bioMérieux), both detecting MS1 spectra fingerprint consisting of most abundant proteins present in a wide array of microorganisms [24–26]. The US Food and Drug Administration (FDA) has issued regulatory approval for using MALDI-TOF mass spectrometry-based platform for routine identification of pathogenic microbes from human specimens in clinical microbiology laboratories [23, 27]. This instrument is coupled with dedicated software and database so it can perform a comparison of the recorded MS1 spectra with the mass spectra of known microorganisms stored in the database. However, MALDI-TOF MS has its limitations and does not allow identification of microorganisms at the species level, nor it performs well when more than one species or strain is present in the sample [28–30]. Furthermore, in order to obtain reliable results, samples have to be cultured on selective agar and a single microbial colony is then used to identify an organism. To bypass time-consuming and selective cultivation stage, culture-independent methods have been developed [17, 31–34]. More recently, there has been growing interest in mass spectrometry based proteomic analyses directly from urine samples, thus skipping the cultivation stage [35, 36]. Ideally, the metaproteomic analysis should be able to provide sufficient numbers of strain-specific peptides useful for microbial identification at the genus, species and even strain-level, and it could also be applied to urine samples containing more than one species, including even potential biomarkers used for non-invasive monitoring of human diseases [37–40].

## Methods

### Urine samples collection and storage

Urine specimens were collected from the Centre for Clinical Microbiology and Hospital Infections, University Hospital Dubrava with only exclusion criteria being antimicrobial therapy. Through the period from October to December 2016 total of 2993 urine specimens were received from patients for whom a urinary culture analysis was requested (Additional file 2: Table S1). The samples were collected from patients according to the instructions for collecting the urine by midstream clean-catch technique [41].

### Urine culture test

The microorganisms were identified by routine microbiology methods [42]. Aliquots made from urine specimens were inoculated onto McConkey agar and blood agar plates using a 1 µl calibrated loop and incubated aerobically at 37 °C from 18 to 48 h, according to the standard operating procedure at the Centre for Clinical Microbiology and Hospital Infections, University Hospital Dubrava. Single colonies were counted to determine the bacterial concentration. Clinically significant infections were considered those with more than 10^5^ CFU/mL.

### Samples for genomics and proteomics analysis

From samples that tested positive (total of 1571) on urine culture test, 16 samples were randomly selected, matching the following criteria: a.) more than 10^5^ CFU/mL and b.) more than 30 ml of urine. All sixteen urine samples (associated with corresponding laboratory reports) were stored at − 20 °C and used for further genomic and proteomic analyses.

## Genomic analysis

### DNA extraction

Frozen samples were thawed at room temperature and homogenised. Bacterial genomic DNA was extracted using the Maxwell 16 Cell DNA Purification Kit on the Maxwell 16 research instrument (Promega, Madison) according to the manufacturer’s instructions. The concentration of DNA was determined using a Nano-Drop spectrophotometer (Shimadzu Biotech).

## 16S rRNA sequencing and bioinformatics analysis

Extracted DNA was sent to Next Generation Sequencing Service Provider (MR DNA, Texas, USA). Sequencing was performed on an Illumina MiSeq platform using paired-end sequencing protocol. Amplicons of the 16S rRNA gene were generated using primers targeting V3 and V4 variable regions of the ribosomal RNA. A 30-cycle PCR reaction was performed using the HotStarTaq Plus Master Mix Kit (Qiagen, USA). Microbiome bioinformatic analysis was performed using QIIME 2 (Quantitative Insights Into Microbial Ecology) software package version 2018.4 [43]. Paired-end raw sequences were demultiplexed and quality filtered using the q2-demux plugin followed by de-noising with DADA2 [44]. First 7 bases of forward and reverse reads were trimmed, forward reads were truncated to 290 bases, and reverse reads to 240 bases. Taxonomy was assigned to obtained amplicon sequence variants using the q2-feature-classifier [45] which relies on classify-sklearn naive Bayes taxonomy classifier and Greengenes v. 13_8 from which 99% OTUs reference sequences were trimmed to variable regions 3 and 4 [46]. Amplicons were analysed using the QIIME 2 (version 2017.4).

## Proteomics

### Sample preparation

For each sample, a homogenized aliquot of 10 ml urine sample was centrifuged at 1000 *g* at room temperature for 1 min (Additional file 1: Figure S1). Insoluble sediment was discarded, and supernatant was transferred to a new tube and centrifuged at 16,000 *g* at 4 °C for 5 min. The supernatant was discarded, and the bacterial pellet was re-suspended in a buffer (25 mM NH_4_HCO_3_, pH 7.8). The pellet was homogenized on vortex and centrifuged at 16,000 g, at 4 °C for 5 min. This procedure was designed to “wash out” mainly excess human cells and it was repeated three times. Proteins were extracted from the bacterial pellet using 100 µL of bacterial protein extraction reagent B-PER (Thermo-Pierce, USA). Following the manufacturer’s protocol, sample was incubated at room temperature for 15 min and subsequently heated at 100 °C in a water bath for 2 min. Insoluble cellular debris was removed by centrifugation at 16,000 *g* at 4 °C for 5 min. Finally, supernatant with soluble proteins contained in B-PER solution was ready for the next step in proteomics sample preparation.

### In solution digestion

Protein sample contained in B-PER (70 µL) was mixed with 2 µL of trypsin solution (1 mg/mL, Merck, Germany). The in-solution digestion was carried out at 37 °C on a thermoshaker (500 rpm) for 18 h (overnight).

### Peptide fractionation

After 18 h of trypsin in-solution digestion, fractionation was performed using the Agilent Bravo automated liquid handling platform (96-channel tip head) and AssayMAP SCX cartridges according to the manufacturer’s instructions, and fractionation protocol (application note 5991-3602EN), SCX cartridges were primed with 400 mM ammonium formate/1% formic acid/25% acetonitrile (ACN), equilibrated with 1% formic acid/25% ACN, loaded with samples, and eluted sequentially using a 40 mM ammonium formate/25% ACN (pH 3.5; 4.0) 40 mM ammonium acetate/25% ACN, (pH 4.5; 5; 5.5) and 100 mM ammonium hydroxide/25% ACN (pH 9.5). From each processed sample, a total of six fractions were collected by chromatography using a pH modulated stepwise elution method.

### MALDI-TOF/TOF mass spectrometry analysis

For sample analysis, 1 µl of 5-mg/mL α-CHCA (α-cyano-4-hydroxycinnamic acid) matrix solution was mixed with 1 µl of each sample fraction (six fractions per sample). From the resulting solution, 1 µl was spotted onto the Opti-TOF MALDI 384 target plate (AB Sciex). After drying at room temperature, spotted samples were analysed using a 4800 Plus MALDI-TOF/TOF mass spectrometer (Applied Biosystems Inc., Foster City, USA) equipped with a 200 Hz, 355 nm Nd: YAG laser. MS spectra were acquired over a mass range of 800–4500 m/z. Peptide fragmentation was performed at collision energy (CID) of 1 kV in positive ion reflection mode, using nitrogen as collision gas. For each sample up to 20 most intense peaks of MS spectra were selected for MS/MS spectra analysis. Approximately 1000 single shots were accumulated from different positions for MS analysis, and 2000 shots spectra were recorded for the subsequent fragment ion spectra. Internal calibration using trypsin autolysis fragments was performed. MS and MS/MS spectra were acquired using the 4000 Series Explorer software v 3.5.3 (AB Sciex).

### Analysis of proteomics data

Mascot (version 2.1. Matrix Science, UK) analysis was carried out to identify peptides and to search for matching proteins in the NCBI “nr” database (20140312) with taxonomy filter set for *Proteobacteria* (11838333 sequences), *Firmicutes* (5487348 sequences) and *Homo sapiens* (276468 sequences). Search parameters for MS and MS/MS database were as follows: parent ion mass tolerances of 0.3 Da and 0.5 Da fragment ion mass tolerance, trypsin digestion with a maximum of one miscleavage per peptide and methionine oxidation as variable modification. Trypsin specificity was set at C-terminal lysine and arginine unless next residue is proline. Qualitative data analysis was performed with MASCOT using a 95% confidence interval, so the significance threshold was adjusted with the false discovery rate below 5%. In Mascot reports a minimum score of 48 was used.

## Results and discussion

### Urine culture test

All samples, which have undergone proteomics and genomics analyses, were benchmarked against standard urine culture test that accompanied all the samples (Additional file 2: Table S2). Among the 16 clinical samples analysed, 13 were classified as monobacterial infections and 3 were classified as polymicrobial (at least two identified uropathogens). Thirteen samples showed presence of Gram-negative and only three to Gram-positive bacteria. Regarding taxonomic diversity of the samples analysed, according to standard tests, there were 7 different bacterial species in total, belonging to 4 respective genera (Additional file 2: Table S3).

### Effect of storage time and temperature on bacteria in urine samples

Guidelines for the collection and storage of urine specimens differ for different diagnostic purposes. This is something we should be aware of. Urine samples should be collected and stored having in mind exact diagnostic procedures to be carried out. In our study, short-term storage (up to 4 weeks) of urines at − 20 °C showed to be a good choice for the preservation of bacteria in collected samples. Long-term storage (for more than 3 months) at − 80 °C led to biomass loss, most likely due to prolonged freezing which caused greater bacterial cell fragility, thus leading to greater extent of cell disruption during centrifugation (unpublished observations).

## Identification of microorganisms using genomics

### 16S rRNA sequencing results

Identification of bacterial taxa is shown in Table 1. Lowest obtainable taxonomic level for which assignment was possible is being shown as a result of genomic identification. Table 1 provides following information: sample number, conventional urine culture result, DNA concentration and 16S rRNA gene sequencing result.

**Table 1:** Identification results based on conventional urine culture and 16S rRNA gene sequencing

N.o.	Urine culture identification	DNA concentration (ng/μL)	16S rRNA sequencing results
UR1	*Klebsiella pneumoniae ESBL*	3.15	100% Enterobacteriaceae
UR2	*Klebsiella oxytoca*	9.14	97% Enterobacteriaceae
UR3	*Klebsiella pneumoniae ESBL*	6.01	90% Enterobacteriaceae; 5.1% Granulicatella; 1.1% *Anaerococcus*
UR4	*Proteus mirabilis*	6.96	97.7% *Proteus*; 1% Enterobacteriaceae
UR5	*Enterococcus faecalis*	16.22	21.8% Pseudomonas; 13.7% *Propionibacterium acnes*; 11% *Lactobacillus helveticus*; 8.1% *Adhaeribacter*; 8% *Acinetobacter*; 5.9% *Staphylococcus*; 4.9% *Stenotrophomonas*; 3.8% *Hydrogenophaga*; 3.6% Erysipelotrichaceae; 3.1% *Corynebacterium*; 3.1% *Cellulomonas*; 2.3% Aerococcus; 2% *Acidovorax*; 1.9% Lachnospiraceae; 1.6% Sphingobium
UR6	*Enterococcus faecalis*	4.93	51.4% *Enterococcus*; 46.5% Enterococcaceae
UR7	*Enterobacter cloaceae ESBL*	1.84	98% Enterobacter; 0.9% Proteus
UR8	*Citrobacter koseri*	15.94	57.5% Citrobacter koseri; 4.5% Bacteroides; 3.7% *Dysgonomonas*; 2.7% Bacteroides; 2.6% Rikenellaceae; 2.3% *Parabacteroides*; 2% Desulfovibrionaceae; 2% Lachnospiraceae; 2% Ruminococcaceae; 1.9% Enterobacteriaceae; 1.3% *Ruminococcus*; 1.3% Erysipelotrichaceae; 1.2% Enterococcus; 1.1% Clostridiales
UR9	*Proteus mirabilis*	0.45	96.7% Proteus; 2.4% Enterobacteriaceae; 1.2% Prevotella
UR10	*Proteus mirabilis*	3.66	97% Proteus; 1.3% Enterobacteriaceae
UR11	*Escherichia coli; Proteus mirabilis ESBL*	9.39	93% Enterobacteriaceae; 3.5% Proteus
UR12	*Proteus mirabilis*	1.73	99.2% Proteus
UR13	*Enterobacter aerogenes*	1.08	75.4% Enterobacteriaceae; 14% Lactobacillus delbrueckii; 4.2% Kluyvera; 4% Enterobacter; 1% *Lactobacillus helveticus*
UR14	*Enterobacter cloacae*	4.14	95.4% Enterobacteriaceae; 1.2% *Clostridium perfringens*; 1% Bifidobacterium pseudolongum
UR15	*Enterobacter cloacae; Enterococcus faecalis; E coli; Proteus mirabilis*	15.17	86.9% Proteus; 7.2% Enterobacteriaceae; 2..2% Enterobacter; 1% Rhodospirillaceae
UR16	*Escherichia coli; Klebsiella pneumoniae*	15..04	91.2% Enterobacteriaceae; 8.6% *Klebsiella*

What stands out in this table is a disparity in taxonomic identification obtained through 16S rRNA gene sequencing—in the majority of cases bacteria were identified on genus level (44%) and family level (56%), while the identification on species level is usually lacking.

It is apparent that *Klebsiella* spp. (UR1-UR3), and *Enterobacter* spp. (UR13-UR14) identifications are difficult to compare due to different levels of taxonomy assignment by the method [47], while there is a significant positive correlation amongst other results for both conventional and genomics methods. A possible explanation for this difficulty might be related to bacterial nomenclature, taxonomy and very high sequence identity. Furthermore, genomic based 16S rRNA analysis was not informative at the genus and/or species level in the family *Enterobacteriaceae* [48]. There was a surprising difference between standard test and genomics results in sample UR 5. Standard urine culture test indicated *Enterococcus faecalis* as a single uropathogen in this sample, while 16S rRNA indicated polymicrobial mixture without *Enterococcus* genus listed. There are two possible explanations for this disparity, one indicating a urine collection sample contamination [49] which would likely cause a genomics test error, and the other being false-positive result of standard culture-based urine test giving a false positive *Enterococcus* result.

## Method for proteomics-based identification of uropathogens

The present study was undertaken to assess the potential of bottom-up proteomics for identification of pathogens directly from the urine samples of patients with UTIs by benchmarking the results obtained against the reference ones (standard urine tests) and using the 16S rRNA gene sequencing—genomics for arbitration in cases where proteomics gives results which differ from the standard urine test.

### Sample preparation

For the proteomic analysis, a minimum concentration of 10^5^ CFU/mL and a volume of 5 mL of fresh urine sample or urine stored in the refrigerator up to 4 weeks were used. In this preliminary study, we investigated and compared the preparation of samples stored at − 20 °C and − 80 °C. We based our decision on the optimal storage temperature of samples on visual inspection of pellets during centrifugation. In the case of urine samples stored at − 80 °C bacterial cells were lost, and the pellet was deemed insufficient for further downstream analysis. On the other hand, samples stored at − 20 °C showed abundant biomass, however, this proved to be a challenge to wash. Reason for this could be cell aggregation, probable auto-aggregation, especially since blood was present in tested samples [32]. Furthermore, good separation of bacterial cells from other materials such as yeast cells, epithelial cells, leukocytes, erythrocytes, mucus, urinary casts, and different types of crystals that can be present in urine depends on centrifugation speed [32, 49]. Moreover, at high-speed the pellet will likely be abundant with cell debris. Consequently, damaged cells will be washed off during the sample preparation process. Pellet volume was identified as an important element that influenced the success of positive protein identification. Microbial biomass had to be visible to the naked eye after washing steps. The obtained pellet biomass can be seen in Additional file 1: Figure S2.

Previous studies had considered the impact of ultra-sonication on microorganisms to improve sample preparation [32, 50, 51]. In our research protein extraction using B-PER worked for both gram-negative and gram-positive bacteria, so there was no need for additional mechanical methods of cell rupture. In reviewed literature, no data was found on the efficiency of protein digestion in the presence of B-PER. We believe that no other group has reported the use of trypsin in the B-PER solution.

### Peptide fractionation

During a preliminary study, we found that the amount of data we could get from one sample spot was insufficient. Thus, to overcome this obstacle we used peptide fractionation. We hypothesised that peptide fractionation would help to enrich the low-abundance peptides (Additional file 1: Figure S4).

## MALDI-TOF/TOF mass spectrometry results

### Protein identifications and data analysis

While BioTyper and Vitek use reference databases to identify and classify the microorganisms according to their mass spectra fingerprint, we relied on peptide ion fragments from MS/MS scans and MASCOT protein search results which were translated into MASCOT based uropathogen identification ranks. For this purpose, we have combined MASCOT score with a peptide count and made a simple Python script that ranks organisms suspected to be in the sample based on probability of their proteins being detected. First step was protein identification of tryptic peptides conducted using MASCOT search engine [52]. This provided us with both score and number of queries matched for proteins belonging to one or more organisms. The Mascot Score is a statistical score for how well the spectra generated match the database protein sequence [52, 53]. Plainly, a higher score indicates a more confident protein match while the number of queries matched indicates the number of spectra that were matched to this protein. Although it is not unusual for a portion of peptides to be scanned multiple times, overall, the greater the score and greater the number of queries matched—greater the probability of a true positive match. Therefore, we have combined these two measures into a “summa score”, simply by summing up all individual peptide scores for a given protein match. Proteins and respective taxa were ordered based on this “summa score” in descending order and highest scoring taxa was taken as most likely uropathogen identification. Table 2 compares the results of this analysis with the standard urine culture test. Summarized report on MASCOT identified bacterial proteins is listed in Additional file 2: Table S4.

**Table 2:** MALDI-TOF/TOF analysis with MASCOT identification of uropathogens

N.o.	Urine culture identification	Mascot identification
UR1	*Klebsiella pneumoniae*	*Klebsiella pneumoniae*
UR2	*Klebsiella oxytoca*	*Klebsiella pneumoniae*
UR3	*Klebsiella pneumoniae*	*Klebsiella pneumoniae*
UR4	*Proteus mirabilis*	*Proteus mirabilis*
UR5	*Enterococcus faecalis*	*Enterococcus faecalis*
UR6	*Enterococcus faecalis*	*Enterococcus faecalis*
UR7	*Enterobacter cloaceae*	*Citrobacter freundii*
UR8	*Citrobacter koseri*	*Citrobacter freundii*
UR9	*Proteus mirabilis*	*Proteus mirabilis*
UR10	*Proteus mirabilis*	*Proteus mirabilis*
UR11	*Escherichia coli; Proteus mirabilis ESBL*	*Escherichia fergusonii*
UR12	*Proteus mirabilis*	*Proteus mirabilis*
UR13	*Enterobacter aerogenes*	*Enterobacter aerogenes*
UR14	*Enterobacter cloacae*	*Klebsiella pneumoniae*
UR15	*Enterobacter cloacae; Enterococcus faecalis; E. coli; Proteus mirabilis*	*Enterococcus faecalis*
UR16	*Escherichia coli;* *Klebsiella pneumoniae*	*Pectobacterium atrosepticum*

The proteins ordered by summa score were listed in Additional file 3: Table S1. Significant minimum MASCOT summa score obtained for all samples was 53, while maximum reported score was 830. A total number of 382 peptides were reported for all 16 samples. Most of these peptides belong to bacterial proteins (71%). Although we expected the majority of proteins belonging to ribosomes, we identified a rather small percentage of ribosomal proteins (8%). In our case proteins with the highest scores, were membrane proteins including outer membrane porin protein C, peptidoglycan-associated lipoprotein (PAL) and murein lipoprotein (MLP). This interesting result might be associated with the usage of the B-PER [54]. Considering all monobacterial samples, direct identifications provided reliable identification for genus *Klebsiella* (3 samples), *Proteus* (4 samples), *Enterococcus* (2 samples), *Enterobacter* (1 sample) and *Citrobacter* (1 sample). Overall, 87% of correlation with standard urine test was obtained with this simple proteomics approach for monobacterial samples.

These results are very encouraging since pathogenic species were correctly identified at the genus level using a relatively small number of identified bacterial proteins per sample, and in the absence of unique peptides. Although our results indicate that proteomics-based identification with a small number of proteins is feasible, high-throughput setup yielding more spectra and retrieving larger fractions of proteomes would be more favourable.

### Microbial identification in polymicrobial cultures

To investigate polymicrobial cultures (UR11, UR15 and UR16), we compared the results obtained from the conventional urine culture, 16S rRNA gene sequencing and proteomics (Additional file 2: Table S5). Our previous experience with MALDI-TOF/TOF mass spectrometer indicated that bacterial identification in polymicrobial urine samples using this platform for proteomics has some limitations. As reported previously by other authors, MALDI-TOF MS identification of polymicrobial cultures directly from urine samples did not provide reliable results [17, 49]. Therefore, bacterial identification at the strain-level is still regarded as a challenge. Some of the underlying factors that compromise this method sensitivity in bacterial identification are: sample impurity substances (human proteins), low abundance of bacterial proteins in the sample [55], insufficient coverage of urinary bacterial species in the databases, shared peptide sequences among proteins from different taxa [38] as well as possibility of generating insufficient level of data by single MS injection per sample [39]. Bottom-up tandem MS accompanied with ever-growing proteomics and genomics databases and data processing through wide range of bioinformatics tools has made polymicrobial identification feasible [30, 36] but it still remains in domain of experimental research and far from clinical practice.

### Human proteins versus contamination

Normal human urine of a healthy individual contains over 2000 proteins [56, 57], while over 5000 proteins can be found when the urinary tract is under inflammation [33]. Due to low protein concentration, urine is a difficult proteomic sample to work with [58].

We recorded 29% of human proteins in our samples, of which 33% were found to be repetitive (Additional file 2: Table S6). The most abundant of these repeated human proteins were classified as haemoglobin subunits (alpha and beta-globin), apolipoprotein and uromodulin. We did not find any evidence of epithelial cells from the urinary or vaginal tract, or any biomarkers.

As can be seen from Additional file 1: Figure S3, first two fractions cover more than 50% of the total number of proteins. Furthermore, Additional file 1: Figure S4 shows a quantitative overview of bacterial and human proteins of each sample. In terms of future work, it would be interesting to consider two-dimensional fractionation to increase bacterial proteome coverage and enhance the ratio of bacterial vs human proteins.

## Limitations and future direction

With regard to the research method, the major limitation identified by this study is a small number of identified proteins per sample. Many proteomic analyses for bacterial identification were limited to monomicrobial specimens with high CFU/mL concentration based on our need to compare results with those of standard urine culture tests, which have own inherent drawbacks. This study lays the groundwork for future research. In the future, a possible direction could be dealing with lower abundant proteins to enhance effectiveness in proteome identification. Switching to a high-throughput platform such as ESI could solve this issue. Furthermore, to increase the number of proteins, a possible solution could be usage of peptide double fractionation or FASP (filter-aided sample preparation) method. To improve bacterial identification, we are developing bioinformatics software based on natural language processing. Urine is clinically underutilized and has a much greater potential in development of non-invasive tests and techniques. Proteomics approach and direct sample analysis have potential to provide us with a broader clinical picture that could bring us closer to precision medicine.

## Conclusion

The main goal of the current study was to establish a procedure for analysis of uropathogens by proteomics, the procedure was tested using MALDI-TOF/TOF mass spectrometry directly from urine specimens. This study has shown that identification of bacteria from a native urine sample, without prior culturing step, depends on storage conditions, sample preparation method, as well as data analysis. Overall, the results of this study demonstrate that mass spectrometry based proteomics can effectively identify different uropathogens from fresh or cold stored, human urine samples directly, without cultivation step. The direct approach was able to provide reliable identification of bacteria at the genus-level in monobacterial samples, despite inherent limitations of mass spectrometry platform used. In case of polymicrobial urine samples, direct approach using the methods here described did not allow for unambiguous identification.

## Supplementary information

**Additional file 1: Figure S1.** Experimental workflow for the identification of uropathogen from a native urine sample. **Figure** **S2.** Images of 16 urine specimens. **Figure** **S3.** Protein content of each fraction as a percentage of the total protein. **Figure** **S4.** Cumulative number of bacterial and human proteins for each sample per fraction.

**Additional file 2: Table S1.** General information about patients. **Table** **S2.** Results of conventional urine culture and urine dipstick analysis for 16 urine samples. **Table** **S3.** Uropathogenic bacteria in urine samples. **Table** **S4.** Summary reports of identified bacterial proteins for each urine sample sorted by “MASCOT summa score”. **Table** **S5.** The comparative view of urine culture, proteomics and genomic results. **Table** **S6.** Identified human proteins ranked by MASCOT score for each urine sample.

**Additional file 3:** Excel tables.

## Data Availability

All the datasets analysed in the current study are available upon reasonable request. There is an archive available at: http://proteinreader.bioinfo.pbf.hr/urine/urine_spectra.zip with the mass spectrometry raw data used.

## Abbreviations

ACN: Acetonitrile
CFU/mL: Colony-forming units per millilitre
LC: Liquid chromatography
MALDI: Matrix-assisted laser desorption/ionization
MS: Mass spectrometry
PMF: Peptide mass fingerprint
TOF: Time of flight
UTI: Urinary tract infection

## References

[1] Foxman B (2010). The epidemiology of urinary tract infection. Nat Rev Urol..

[2] Dason S, Dason JT, Kapoor A (2011). Guidelines for the diagnosis and management of recurrent urinary tract infection in women. J Can Urol Assoc..

[3] Bouchillon SK, Badal RE, Hoban DJ, Hawser SP (2013). Antimicrobial susceptibility of inpatient urinary tract isolates of gram-negative bacilli in the United States: results from the Study for Monitoring Antimicrobial Resistance Trends (SMART) Program: 2009–2011. Clin Ther.

[4] Brubaker L, Wolfe AJ (2015). The new world of the urinary microbiome in women linda. Am J Obstet Gynecol.

[5] Koves B, Wullt B (2016). The roles of the host and the pathogens in urinary tract infections. Eur Urol Suppl..

[6] Schmiemann G, Kniehl E, Gebhardt K, Matejczyk MM, Hummers-Pradier E (2010). The diagnosis of urinary tract infection: a systematic review. Dtsch Ärzteblatt Int..

[7] Hilt EE, McKinley K, Pearce MM, Rosenfeld AB, Zilliox MJ, Mueller ER (2014). Urine is not sterile: use of enhanced urine culture techniques to detect resident bacterial flora in the adult female bladder. J Clin Microbiol.

[8] Wolfe AJ, Brubaker L (2015). “Sterile Urine” and the presence of bacteria. Eur Urol.

[9] Ferreira L, Sánchez-Juanes F, Muñoz-Bellido JL, González-Buitrago JM (2011). Rapid method for direct identification of bacteria in urine and blood culture samples by matrix-assisted laser desorption ionization time-of-flight mass spectrometry: intact cell vs extraction method. Clin Microbiol Infect..

[10] Burd EM, Kehl KS (2011). A critical appraisal of the role of the clinical microbiology laboratory in the diagnosis of urinary tract infections. J Clin Microbiol.

[11] Cavagnolo R (1995). Evaluation of incubation times for urine cultures. J Clin Microbiol.

[12] Brilha S, Proença H, Cristino JM, Hänscheid T (2010). Use of flow cytometry (Sysmex UF-100) to screen for positive urine cultures: in search for the ideal cut-off. Clin Chem Lab Med.

[13] Delanghe J, Speeckaert M (2014). Preanalytical requirements of urinalysis. Biochem Medica..

[14] Davenport M, Mach KE, Shortliffe LMD, Banaei N, Wang H, Liao JC (2017). New and developing diagnostic technologies for urinary tract infections. Nat Rev Urol..

[15] Franco-Duarte R, Černáková L, Kadam S, Kaushik KS, Salehi B, Bevilacqua A (2019). Advances in chemical and biological methods to identify microorganisms—from past to present. Microorganisms..

[16] Singhal N, Kumar M, Kanaujia PK, Virdi JS (2015). MALDI-TOF mass spectrometry: an emerging technology for microbial identification and diagnosis. Front Microbiol..

[17] Zboromyrska Y, Rubio E, Alejo I, Vergara A, Mons A, Campo I (2016). Development of a new protocol for rapid bacterial identification and susceptibility testing directly from urine samples. Clin Microbiol Infect.

[18] De Rosa R, Grosso S, Lorenzi G, Bruschetta G, Camporese A (2018). Evaluation of the new Sysmex UF-5000 fluorescence flow cytometry analyser for ruling out bacterial urinary tract infection and for prediction of Gram negative bacteria in urine cultures. Clin Chim Acta.

[19] Van Der Zee A, Roorda L, Bosman G, Ossewaarde JM (2016). Molecular diagnosis of urinary tract infections by semi-quantitative detection of uropathogens in a routine clinical hospital setting. PLoS ONE..

[20] Mignard S, Flandrois JP (2006). 16S rRNA sequencing in routine bacterial identification: a 30-month experiment. J Microbiol Methods.

[21] Sabat AJ, Van Zanten E, Akkerboom V, Wisselink G, Van Slochteren K, De Boer RF (2017). Targeted next-generation sequencing of the 16S-23S rRNA region for culture-independent bacterial identification-increased discrimination of closely related species. Sci Rep..

[22] Mann M, Hendrickson RC, Pandey A (2001). Analysis of proteins and proteomes by mass spectrometry. Annu Rev Biochem.

[23] Cheng K, Chui H, Domish L, Hernandez D, Wang G (2016). Recent development of mass spectrometry and proteomics applications in identification and typing of bacteria. Proteomics Clin Appl..

[24] Lévesque S, Dufresne PJ, Soualhine H, Domingo MC, Bekal S, Lefebvre B (2015). A side by side comparison of Bruker Biotyper and VITEK MS: utility of MALDI-TOF MS technology for microorganism identification in a public health reference laboratory. PLoS ONE.

[25] Jang KS, Kim YH (2018). Rapid and robust MALDI-TOF MS techniques for microbial identification: a brief overview of their diverse applications. J Microbiol..

[26] Li Y, Shan M, Zhu Z, Mao X, Yan M, Chen Y (2019). Application of MALDI-TOF MS to rapid identification of anaerobic bacteria. BMC Infect Dis..

[27] Lathrop JT, Jeffery DA, Shea YR, Scholl PF, Chan MM (2016). US food and drug administration perspectives on clinical mass spectrometry. Clin Chem.

[28] Patel R (2015). MALDI-TOF MS for the diagnosis of infectious diseases. Clin Chem.

[29] Henzel WJ, Watanabe C, Stults JT (2003). Protein identification: the origins of peptide mass fingerprinting. J Am Soc Mass Spectrom.

[30] Karlsson R, Gonzales-siles L, Boulund F, Svensson-stadler L, Skovbjerg S, Karlsson A (2015). Proteotyping: proteomic characterization, classification and identification of microorganisms—a prospectus. Syst Appl Microbiol.

[31] Veron L, Mailler S, Girard V, Muller BH, L’Hostis G, Ducruix C (2015). Rapid urine preparation prior to identification of uropathogens by MALDI-TOF MS. Eur J Clin Microbiol Infect Dis.

[32] Kitagawa K, Shigemura K, Onuma KI, Nishida M, Fujiwara M, Kobayashi S (2018). Improved bacterial identification directly from urine samples with matrix-assisted laser desorption/ionization time-of-flight mass spectrometry. J Clin Lab Anal..

[33] Yu Y, Sikorski P, Bowman-Gholston C, Cacciabeve N, Nelson KE, Pieper R (2015). Diagnosing inflammation and infection in the urinary system via proteomics. J Transl Med..

[34] Íñigo M, Coello A, Fernández-Rivas G, Rivaya B, Hidalgo J, Quesada MD (2016). Direct identification of urinary tract pathogens from urine samples, combining urine screening methods and matrix-assisted laser desorption ionization—time of flight mass spectrometry. J Clin Microbiol.

[35] Shao W, Zhang M, Lam H, Lau SCK (2015). A peptide identification-free, genome sequence-independent shotgun proteomics workflow for strain-level bacterial differentiation. Sci Rep..

[36] Boulund F, Karlsson R, Gonzales-Siles L, Johnning A, Karami N, AL-Bayati O (2017). Typing and characterization of bacteria using bottom-up tandem mass spectrometry proteomics. Mol Cell Proteomics..

[37] Boulund F, Karlsson R, Gonzales-Siles L, Johnning A, Karami N, AL-Bayati O, et al. Typing and Characterization of Bacteria Using Bottom-up Tandem Mass Spectrometry Proteomics. Mol Cell Proteomics. 2017;16(6):1052–63. http://www.mcponline.org/lookup/doi/10.1074/mcp.M116.061721. 10.1074/mcp.M116.061721 PMC5461537 28420677

[38] Alves G, Wang G, Ogurtsov AY, Drake SK, Gucek M, Sacks DB (2018). Rapid classification and identification of multiple high-resolution tandem mass spectrometry. J Am Soc Mass Spectrom.

[39] Karlsson R, Davidson M, Svensson-Stadler L, Karlsson A, Olesen K, Carlsohn E (2012). Strain-level typing and identification of bacteria using mass spectrometry-based proteomics. J Proteome Res..

[40] Yu Y, Sikorski P, Smith M, Bowman-Gholston C, Cacciabeve N, Nelson KE (2017). Comprehensive metaproteomic analyses of urine in the presence and absence of neutrophil-associated inflammation in the urinary tract. Theranostics..

[41] Lifshitz E, Kramer L (2000). Outpatient urine culture: does collection technique matter?. Arch Intern Med.

[42] Price TK, Dune T, Hilt EE, Thomas-White KJ, Kliethermes S, Brincat C (2016). The clinical urine culture: enhanced techniques improve detection of clinically relevant microorganisms. J Clin Microbiol.

[43] Bolyen E, Rideout JR, Dillon MR, Bokulich NA, Abnet CC, Al-Ghalith GA (2019). Reproducible, interactive, scalable and extensible microbiome data science using QIIME 2. Nat Biotechnol.

[44] Callahan BJ, McMurdie PJ, Rosen MJ, Han AW, Johnson AJA, Holmes SP (2016). DADA2: high-resolution sample inference from Illumina amplicon data. Nat Methods.

[45] Bokulich NA, Kaehler BD, Rideout JR, Dillon M, Bolyen E, Knight R (2018). Optimizing taxonomic classification of marker-gene amplicon sequences with QIIME 2′s q2-feature-classifier plugin. Microbiome..

[46] McDonald D, Price MN, Goodrich J, Nawrocki EP, Desantis TZ, Probst A (2012). An improved Greengenes taxonomy with explicit ranks for ecological and evolutionary analyses of bacteria and archaea. ISME J.

[47] Kotaskova I, Obrucova H, Malisova B, Videnska P, Zwinsova B, Peroutkova T (2019). Molecular techniques complement culture-based assessment of bacteria composition in mixed biofilms of urinary tract catheter-related samples. Front Microbiol..

[48] Naum M, Brown EW, Mason-Gamer RJ (2008). Is 16S rDNA a reliable phylogenetic marker to characterize relationships below the family level in the enterobacteriaceae?. J Mol Evol.

[49] Lough ME, Shradar E, Hsieh C, Hedlin H (2019). Contamination in adult midstream clean-catch urine cultures in the emergency department: a randomized controlled trial. J Emerg Nurs..

[50] Huang B, Zhang L, Zhang W, Liao K, Zhang S, Zhang Z (2017). Direct detection and identification of bacterial pathogens from urine with optimized specimen processing and enhanced testing algorithm. J Clin Microbiol.

[51] Sigdel TK, Nicora CD, Hsieh SC, Dai H, Qian WJ, Camp DG (2014). Optimization for peptide sample preparation for urine peptidomics. Clin Proteomics.

[52] Cottrell JS (2011). Protein identification using MS/MS data. J Proteomics..

[53] Perkins DN, Pappin DJ, Creasy DM, Cottrell JS (1999). Probability-based protein identification by searching sequence databases using mass spectrometry data. Electrophoresis..

[54] Zhang X, Li L, Mayne J, Ning Z, Stintzi A, Figeys D (2018). Assessing the impact of protein extraction methods for human gut metaproteomics. J Proteomics..

[55] Harpole M, Davis J, Espina V (2016). Current state of the art for enhancing urine biomarker discovery. Expert Rev Proteomics..

[56] Koenig T, Menze BH, Kirchner M, Monigatti F, Parker KC, Patterson T (2008). Robust prediction of the MASCOT score for an improved quality assessment in mass spectrometric proteomics. J Proteome Res.

[57] Adachi J, Kumar C, Zhang Y, Olsen JV, Mann M (2006). The human urinary proteome contains more than 1500 proteins, including a large proportion of membrane proteins. Genome Biol..

[58] Lin L, Yu Q, Zheng J, Cai Z, Tian R (2018). Fast quantitative urinary proteomic profiling workflow for biomarker discovery in kidney cancer. Clin Proteomics..

