# Supplementary material for: Identification of pathogens from native urine samples by MALDI-TOF/TOF tandem mass spectrometry
Source: Clin Proteomics. 2020 Jun 23;17:25. doi: 10.1186/s12014-020-09289-4 (PMC7310424; doi:10.1186/s12014-020-09289-4)
Supplement: Supplementary file 1 — Additional file 1: Figure S1. Experimental workflow for the identification of uropathogen from a native urine sample. Figure S2. Images of 16 urine specimens. Figure S3. Protein content of each fraction as a percentage of the total protein. Figure S4. Cumulative number of bacterial and human proteins for each sample per fraction. [file 12014_2020_9289_MOESM1_ESM.docx]

**Supporting information data**


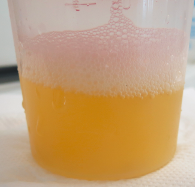


**Additional file 1**

**Urine sample** (10 mL)

**Bacterial cells enrichment**
centrifugation (1 min 1000 *g*, RT)

**Washing steps - with buffer**
centrifugation (16000 *g* for 5 min, 4°C)

**Protein extraction**
B-PER protocol

**In-solution digestion**
thermoshaker (18 h, 37°C, 500 rpm)

**Peptide fractionation**automated liquid handling platform Bravo

**Mass spectrometry analysis MALDI TOF/TOF**
- MS1 (precursor spectra)
- MS2 (fragmentation spectra)

**Bioinformatic data analysis**MASCOT search engine

**Identification of bacteria**

**Figure S1: Experimental workflow for the identification of uropathogen from a native urine sample.** The workflow covers sample preparation, sample fractionation, mass spectrometry analysis and data analysis.

Sample UR1

A)
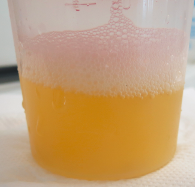
 B)
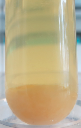
 C)
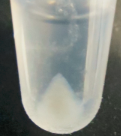


Sample UR2

1.
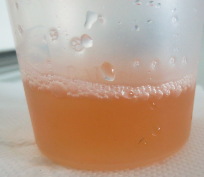
 B)
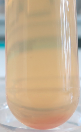
 C)
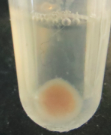


Sample UR3

1.
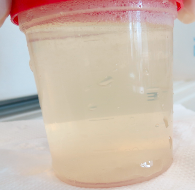
 B)
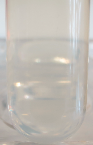
 C)
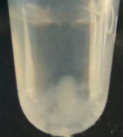


Sample UR4

1.
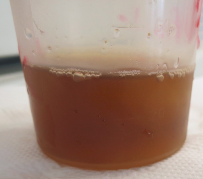
 B)
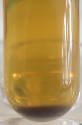
 C)
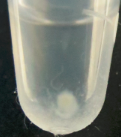


Sample UR5

1.
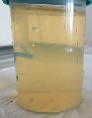
 B)
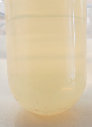
 C)
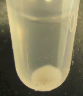


Sample UR6

1.
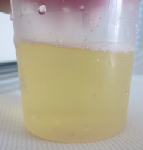
 B)
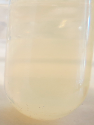
 C)
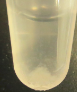


Sample UR7

1.
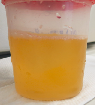
 B)
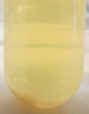
 C)
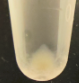


Sample UR8

1.
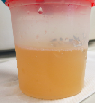
 B)
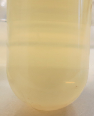
 C)
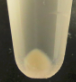


Sample UR9

1.
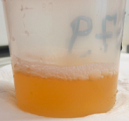
 B)
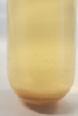
 C)
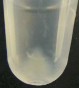


Sample UR10

1.
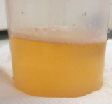
 B)
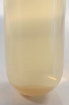
 C)
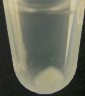


Sample UR11

1.
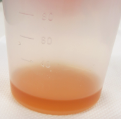
 B)
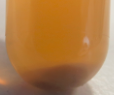
 C)
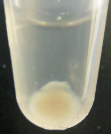


Sample UR12

1.
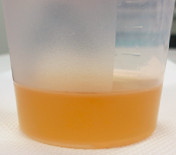
 B)
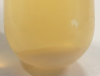
 C)
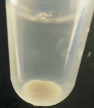


Sample UR13

1.
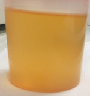
 B)
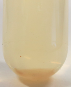
 C)
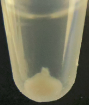


Sample UR14

1.
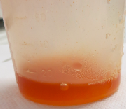
 B)
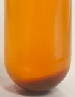
 C)
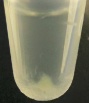


Sample UR15

1.
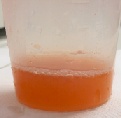
 B)
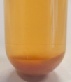
 C)
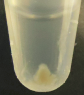


Sample UR16

1.
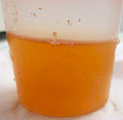
 B)
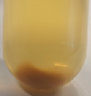
 C)
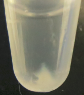


**Figure S2: Images of 16 urine specimens.**(A) Urine sample in container, (B) Urinary pellet (before washing step),
(C) Urinary cell pellet in B-PER.

**Figure S3: Protein content of each fraction as a percentage of the total protein.**

**Figure S4: Cumulative number of bacterial and human proteins for each sample per fraction.**
