# Supplementary material for: Identification of pathogens from native urine samples by MALDI-TOF/TOF tandem mass spectrometry
Source: Clin Proteomics. 2020 Jun 23;17:25. doi: 10.1186/s12014-020-09289-4 (PMC7310424; doi:10.1186/s12014-020-09289-4)
Supplement: Supplementary file 2 — Additional file 2: Table S1. General information about patients. Table S2. Results of conventional urine culture and urine dipstick analysis for 16 urine samples. Table S3. Uropathogenic bacteria in urine samples. Table S4. Summary reports of identified bacterial proteins for each urine sample sorted by “MASCOT summa score”. Table S5. The comparative view of urine culture, proteomics and genomic results. Table S6. Identified human proteins ranked by MASCOT score for each urine sample. [file 12014_2020_9289_MOESM2_ESM.docx]

**Supporting information data**

**Additional file 2**

**Table S1: General information about patients.**

| Characteristics |  | |
| --- | --- | --- |
| Age | Mean | 66.8 |
|  | Range | 4-82 |
| Gender | Male | 5 (31%) |
|  | Female | 11 (69%) |
|  | Outpatients | 15 (94%) |
|  | Urine catheter | 4 (25%) |

**Table S2: Results of conventional urine culture and urine dipstick analysis for 16 urine samples.**

| N.o. | Species (urine culture) | | CFU/mL | | Leukocyte | | Nitrite | | Urobilinogen | | Protein | | pH | | Blood | |  |
| --- | --- | --- | --- | --- | --- | --- | --- | --- | --- | --- | --- | --- | --- | --- | --- | --- | --- |
| UR1 | | *Klebsiella pneumoniae ESBL* | | > 100000 | | ++ | | - | | - | | +++ | | 8,5 | | - | |
| UR2 | | *Klebsiella oxytoca* | | > 100000 | | - | | + | | - | | ++ | | 8,5 | | - | |
| UR3 | | *Klebsiella pneumoniae ESBL* | | > 100000 | | + | | + | | - | | + | | 8 | | +++ | |
| UR4 | | *Proteus mirabilis* | | > 100000 | | ++ | | + | | - | | ++ | | 8 | | +++ | |
| UR5 | | *Enterococcus faecalis* | | > 100000 | | - | | - | | -- | | - | | 7 | | - | |
| UR6 | | *Enterococcus faecalis* | | > 100000 | | - | | - | | - | | - | | 7,5 | | + | |
| UR7 | | *Enterobacter cloaceae ESBL* | | > 100000 | | ++ | | - | | --- | | ++ | | 8 | | +++ | |
| UR8 | | *Citrobacter koseri* | | > 100000 | | - | | - | | - | | + | | 6 | | - | |
| UR9 | | *Proteus mirabilis* | | > 100000 | | - | | - | | - | | ++ | | 8,5 | | - | |
| UR10 | | *Proteus mirabilis* | | > 100000 | | ++ | | ++ | | - | | - | | 8,5 | | - | |
| UR11 | | *Escherichia coli; Proteus mirabilis ESBL* | | > 100000; > 100000; | | - | | - | | - | | + | | 8 | | ++ | |
| UR12 | | *Proteus mirabilis* | | >100000 | | ++ | | - | | - | | +++ | | 7 | | - | |
| UR13 | | *Enterobacter aerogenes* | | > 100000 | | ++ | | - | | - | | - | | 5 | | - | |
| UR14 | | *Enterobacter cloacae* | | > 100000 | | ++ | | ++ | | - | | ++ | | 6,5 | | +++ | |
| UR15 | | *Enterobacter cloacae; Enterococcus faecalis; E coli; Proteus mirabilis* | | 10000;  > 10000 < 100000; 100000; > 100000; | | - | | - | | - | | ++ | | 7,5 | | +++ | |
| UR16 | | *Escherichia coli; Klebsiella pneumoniae* | | >100000; >100000 | | ++ | | - | | - | | + | | 6 | | + | |

**Table S3: Uropathogenic bacteria in urine samples (standard urine culture)**

1. Monomicrobial urine samples

| **Bacterial species** | **Sample** | **Number of samples** |
| --- | --- | --- |
| *Citrobacter koseri* | UR8 | 1 |
| *Enterobacter aerogenes* | UR13 | 1 |
| *Enterobacter cloaceae* | UR7, UR14 | 2 |
| *Enterococcus faecalis* | UR5, UR6 | 2 |
| *Klebsiella oxytoca* | UR2 | 1 |
| *Klebsiella pneumoniae* | UR1, UR3 | 2 |
| *Proteus mirabilis* | UR4, UR9, UR10, UR12 | 4 |

1. Polymicrobial urine samples

| **Bacterial species** | **Sample** | **Number of samples** |
| --- | --- | --- |
| *Enterococcus faecalis* | UR15 | 1 |
| *Escherichia coli* | UR11, UR15, UR16 | 3 |
| *Klebsiella pneumoniae* | UR16 | 1 |
| *Proteus mirabilis* | UR11, UR15 | 2 |

**Table S4: Summary reports of identified bacterial proteins for each urine sample sorted by “MASCOT summa score”.**

***Sample UR1***

|  | Accession | Protein_description | Σ Peptides | | MW (Da) | | ∑ Score | | Bacteria | |  |
| --- | --- | --- | --- | --- | --- | --- | --- | --- | --- | --- | --- |
| 1 | YP_002920498.1 | outer membrane porin protein C | | 4 | | 40008 | | 830 | | ***Klebsiella pneumoniae*** | |
| 2 | YP_001335792.1 | murein lipoprotein | | 4 | | 8381 | | 483 | | *Klebsiella pneumoniae* | |
| 3 | ACM07444.1 | OmpK36 porin | | 1 | | 39854 | | 274 | | *Klebsiella pneumoniae* | |
| 4 | 1006243A | lipoprotein mutant | | 1 | | 8265 | | 233 | | *Escherichia coli* | |
| 5 | YP_001334652.1 | outer membrane protein A | | 2 | | 38021 | | 203 | | *Klebsiella pneumoniae* | |
| 6 | WP_004133882.1 | outer membrane porin protein C | | 1 | | 40676 | | 198 | | *Klebsiella oxytoca* | |
| 7 | WP_016189672.1 | pyruvate formate-lyase | | 1 | | 85234 | | 147 | | *Erwinia tracheiphila* | |
| 8 | YP_001334597.1 | formate acetyltransferase 1 | | 1 | | 85092 | | 144 | | *Klebsiella pneumoniae* | |
| 9 | 2K0L_A | Chain A, Nmr Structure Of The Transmembrane Domain Of The Outer Membrane Protein A | | 1 | | 23355 | | 141 | | *Klebsiella pneumoniae* | |
| 10 | YP_007872516.1 | outer membrane porin protein C | | 1 | | 40486 | | 133 | | *Raoultella ornithinolytica* | |
| 11 | WP_016809062.1 | outer membrane porin protein C | | 1 | | / | | 102 | | *Klebsiella oxytoca* | |
| 12 | P02938.1 | RecName: Full=Major outer membrane lipoprotein; AltName: Full=Murein-lipoprotein; Flags: Precursor | | 1 | | 8234 | | 100 | | *Serratia marcescens* | |
| 13 | AAD32649.1 | EF-Tu | | 1 | | 39423 | | 96 | | *Coxiella burnetii* | |
| 14 | YP_007504946.1 | Pyruvate formate-lyase | | 1 | | 85227 | | 93 | | *Morganella morganii* | |
| 15 | WP_006820044.1 | outer membrane porin protein C | | 1 | | / | | 77 | | *Yokenella regensburgei* | |
| 16 | WP_006707774.1 | murein lipoprotein | | 1 | | 8805 | | 75 | | *Candidatus Regiella insecticola* | |
| 17 | ABS84845.1 | translation elongation factor Tu | | 1 | | 21970 | | 73 | | *Bacillus subtilis* | |
| 18 | NP_872680.1 | elongation factor Tu | | 1 | | 43400 | | 73 | | *Haemophilus ducreyi* | |
| 19 | NP_299905.1 | elongation factor Tu | | 1 | | 42849 | | 70 | | *Xylella fastidiosa* | |
| 20 | YP_001453698.1 | outer membrane protein F | | 1 | | / | | 67 | | *Citrobacter koseri* | |
| 21 | NP_667815.1 | elongation factor Tu | | 1 | | / | | 67 | | *Yersinia pestis* | |
| 22 | P09146.1 | RecName: Full=Outer membrane protein A; Flags: Precursor | | 1 | | 37552 | | 67 | | *Klebsiella aerogenes* | |
| 23 | YP_003755150.1 | translation elongation factor Tu | | 1 | | / | | 65 | | *Hyphomicrobium denitrificans* | |
| 24 | YP_001334418.1 | peptidoglycan-associated outer membrane lipoprotein | | 1 | | 18851 | | 64 | | *Klebsiella pneumoniae* | |
| 25 | YP_004393584.1 | major outer membrane protein OmpAII | | 1 | | / | | 64 | | *Aeromonas veronii* | |
| 26 | NP_455305.1 | peptidoglycan-associated lipoprotein | | 1 | | 18853 | | 64 | | *Salmonella enterica* | |
| 27 | NP_669338.1 | bifunctional acetaldehyde-CoA/alcohol dehydrogenase | | 1 | | 96527 | | 64 | | *Yersinia pestis* | |
| 28 | YP_001335857.1 | bifunctional acetaldehyde-CoA/alcohol dehydrogenase | | 1 | | 95834 | | 62 | | *Klebsiella pneumoniae* | |

***Sample UR2***

|  | Accession | Protein_description | Σ Peptides | MW (Da) | ∑ Score | Bacteria |
| --- | --- | --- | --- | --- | --- | --- |
| 1 | YP_001338507.1 | molecular chaperone | 3 | 21493 | 372 | *Klebsiella pneumoniae* |
| 2 | YP_001335792.1 | murein lipoprotein | 2 | 8381 | 289 | *Klebsiella pneumoniae* |
| 3 | WP_000350438.1 | heat shock protein Hsp20 | 2 | 21465 | 253 | *Escherichia coli* |
| 4 | 1006243A | lipoprotein mutant | 1 | 8265 | 227 | *Escherichia coli* |
| 5 | WP_004103993.1 | outer membrane porin protein C | 1 | 40047 | 196 | ***Klebsiella oxytoca*** |
| 6 | EWF64901.1 | outer membrane protein C | 1 | 40621 | 185 | *Klebsiella oxytoca* |
| 7 | WP_004133882.1 | outer membrane porin protein C | 1 | 40676 | 174 | *Klebsiella oxytoca* |
| 8 | YP_002920498.1 | outer membrane porin protein C | 1 | 40008 | 159 | *Klebsiella pneumoniae* |
| 9 | YP_005018909.1 | peptidoglycan-associated outer membrane lipoprotein | 1 | 18833 | 116 | *Klebsiella oxytoca* |
| 10 | WP_016809062.1 | outer membrane porin protein C | 1 | 41545 | 110 | *Klebsiella oxytoca* |
| 11 | YP_001334652.1 | outer membrane protein A | 1 | 38021 | 98 | *Klebsiella pneumoniae* |
| 12 | P09146.1 | RecName: Full=Outer membrane protein A; Flags: Precursor | 1 | 37552 | 98 | *Klebsiella aerogenes* |
| 13 | YP_004393584.1 | major outer membrane protein OmpAII | 1 | / | 95 | *Aeromonas veronii* |
| 14 | ABM66811.1 | OmpE36 | 1 | 40538 | 86 | *Enterobacter aerogenes* |
| 15 | WP_006820044.1 | outer membrane porin protein C | 1 | 40792 | 81 | *Yokenella regensburgei* |
| 16 | YP_005016763.1 | hypothetical protein KOX_03925 | 1 | 15018 | 76 | *Klebsiella oxytoca* |
| 17 | YP_001175110.1 | 30S ribosomal protein S6 | 1 | 15096 | 74 | *Enterobacter sp.* |
| 18 | AAL56004.1 | GroEL | 1 | 52000 | 73 | *Escherichia coli* |
| 19 | YP_001453698.1 | outer membrane protein F | 1 | / | 70 | *Citrobacter koseri* |
| 20 | YP_004517370.1 | thioredoxin | 1 | 11419 | 65 | *Desulfotomaculum kuznetsovii* |
| 21 | YP_960652.1 | heat shock protein Hsp20 | 1 | 21458 | 62 | *Marinobacter aquaeolei* |
| 22 | P02938.1 | RecName: Full=Major outer membrane lipoprotein; AltName: Full=Murein-lipoprotein; Flags: Precursor | 1 | 8234 | 62 | *Serratia marcescens* |

***Sample UR3***

|  | Accession | Protein_description | Σ Peptides | | MW (Da) | | ∑ Score | | Bacteria | |  |
| --- | --- | --- | --- | --- | --- | --- | --- | --- | --- | --- | --- |
| 1 | YP_001335857.1 | bifunctional acetaldehyde-CoA/alcohol dehydrogenase | | 3 | | 95834 | | 308 | | ***Klebsiella pneumoniae*** | |
| 2 | YP_001335792.1 | murein lipoprotein | | 1 | | 8381 | | 187 | | *Klebsiella pneumoniae* | |
| 3 | 1006243A | lipoprotein mutant | | 1 | | 8265 | | 187 | | *Escherichia coli* | |
| 4 | WP_006777698.1 | major outer membrane lipoprotein | | 1 | | 11702 | | 142 | | *Salmonella enterica* | |
| 5 | YP_001177024.1 | bifunctional acetaldehyde-CoA/alcohol dehydrogenase | | 1 | | 96203 | | 74 | | *Enterobacter sp.* | |
| 6 | ERE53009.1 | 30S ribosomal protein S2, partial | | 1 | | 25562 | | 67 | | *Enterococcus gallinarum* | |
| 7 | BAA32342.1 | ribosomal protein S2 | | 1 | | 31289 | | 67 | | *Pseudomonas aeruginosa* | |
| 8 | YP_001334860.1 | glyceraldehyde-3-phosphate dehydrogenase | | 1 | | 35449 | | 65 | | *Klebsiella pneumoniae* | |
| 9 | YP_007344897.1 | alcohol dehydrogenase, class IV | | 1 | | 95701 | | 64 | | *Serratia marcescens* | |

***Sample UR4***

|  | Accession | Protein_description | Σ Peptides | | MW (Da) | | ∑ Score | | Bacteria | |  |
| --- | --- | --- | --- | --- | --- | --- | --- | --- | --- | --- | --- |
| 1 | WP_004244438.1 | peptidoglycan-associated outer membrane lipoprotein | | 1 | | 19482 | | 263 | | ***Proteus mirabilis*** | |
| 2 | YP_002150530.1 | outer membrane porin | | 2 | | 40796 | | 185 | | *Proteus mirabilis* | |
| 3 | YP_002150547.1 | outer membrane protein A | | 2 | | 38994 | | 146 | | *Proteus mirabilis* | |
| 4 | WP_017827341.1 | outer membrane porin protein C | | 1 | | 41648 | | 106 | | *Proteus mirabilis* | |
| 5 | YP_001334418.1 | peptidoglycan-associated outer membrane lipoprotein | | 1 | | 18851 | | 89 | | *Klebsiella pneumoniae* | |
| 6 | NP_455305.1 | peptidoglycan-associated lipoprotein | | 1 | | 18853 | | 89 | | *Salmonella enterica* | |
| 7 | YP_002987255.1 | porin Gram-negative type | | 1 | | 39618 | | 71 | | *Dickeya dadantii* | |
| 8 | WP_022633981.1 | Outer membrane porin 1a | | 1 | | 40140 | | 69 | | *Dickeya* | |
| 9 | WP_004256113.1 | membrane protein | | 1 | | 39141 | | 69 | | *Providencia rettgeri* | |
| 10 | ADO64273.1 | porin 1 | | 1 | | 41193 | | 69 | | *Providencia stuartii* | |

***Sample UR5***

|  | Accession | Protein_description | Σ Peptides | MW (Da) | ∑ Score | Bacteria |  |
| --- | --- | --- | --- | --- | --- | --- | --- |
| 1 | NP_815088.1 | cold-shock domain-contain protein | 3 | 7181 | 267 | ***Enterococcus faecalis*** | |
| 2 | NP_814901.1 | 50S ribosomal protein L31 | 2 | 10119 | 240 | *Enterococcus faecalis* | |
| 3 | NP_814526.1 | cold shock domain-contain protein | 1 | 7109 | 136 | *Enterococcus faecalis* | |
| 4 | NP_814417.1 | hypothetical protein EF0665 | 2 | 6666 | 132 | *Enterococcus faecalis* | |
| 5 | BAI53110.1 | elongation factor Tu | 1 | 28690 | 91 | *Carnobacterium maltaromaticum* | |
| 6 | AAF17305.1 | putative elongation factor Tu | 1 | 27433 | 91 | *Enterococcus avium* | |
| 7 | ABS84849.1 | translation elongation factor Tu | 1 | 21843 | 91 | *Serratia rubidaea* | |
| 8 | NP_814237.1 | OsmC/Ohr family protein | 1 | 14371 | 90 | *Enterococcus faecalis* | |
| 9 | WP_016173398.1 | general stress protein | 1 | 22194 | 86 | *Enterococcus dispar* | |
| 10 | NP_813885.1 | gls24 protein | 1 | 20540 | 86 | *Enterococcus faecalis* | |
| 11 | NP_267752.1 | 50S ribosomal protein L31 | 1 | / | 83 | *Lactococcus lactis* | |
| 12 | EUJ37103.1 | cold-shock DNA-binding domain-containing protein | 1 | 7459 | 74 | *Brochothrix thermosphacta* | |
| 13 | WP_010765440.1 | hypothetical protein | 1 | 7260 | 70 | *Enterococcus moraviensis* | |
| 14 | EUK53620.1 | 50S ribosomal protein L31 type B | 1 | / | 68 | *Staphylococcus aureus* | |
| 15 | NP_372644.1 | 50S ribosomal protein L31 | 1 | / | 68 | *Staphylococcus aureus* | |
| 16 | NP_815637.1 | enolase | 1 | 46482 | 67 | *Enterococcus faecalis* | |
| 17 | NP_816504.1 | acyl carrier protein | 1 | 8610 | 67 | *Enterococcus faecalis* | |
| 18 | NP_816831.1 | Dps family protein | 1 | 17877 | 67 | *Enterococcus faecalis* | |
| 19 | WP_002568043.1 | cold shock protein | 1 | 7270 | 66 | *Clostridium* | |
| 20 | WP_002586010.1 | cold-shock protein | 1 | 7305 | 66 | *Clostridium* | |

***Sample UR6***

|  | Accession | Protein_description | Σ Peptides | MW (Da) | ∑ Score | Bacteria |
| --- | --- | --- | --- | --- | --- | --- |
| 1 | NP_814526.1 | cold-shock DNA-binding domain-containing protein | 4 | 7109 | 497 | ***Enterococcus faecalis*** |
| 2 | NP_815088.1 | cold-shock domain-contain protein | 2 | 7181 | 142 | *Enterococcus faecalis* |
| 3 | AAC80242.1 | major cold-shock protein, partial | 1 | 4967 | 88 | *Enterococcus faecalis* |

***Sample UR7***

|  | Accession | Protein_description | Σ Peptides | | MW (Da) | | ∑ Score | | Bacteria | |  |
| --- | --- | --- | --- | --- | --- | --- | --- | --- | --- | --- | --- |
| 1 | P24016.1 | RecName: Full=Outer membrane protein A; AltName: Full=Outer membrane protein II | | 3 | | 25648 | | 283 | | *Citrobacter freundii* | |
| 2 | YP_001335792.1 | murein lipoprotein | | 1 | | 8381 | | 208 | | *Klebsiella pneumoniae* | |
| 3 | 1006243A | lipoprotein mutant | | 1 | | 8265 | | 208 | | *Escherichia coli* | |
| 4 | YP_049963.1 | major outer membrane lipoprotein | | 1 | | 8396 | | 148 | | *Pectobacterium atrosepticum* | |
| 5 | AHF77098.1 | Major outer membrane lipoprotein | | 1 | | 8327 | | 148 | | *Sodalis sp.* | |
| 6 | WP_007664753.1 | Outer membrane protein A precursor | | 1 | | 38294 | | 113 | | *Cronobacter condimenti* | |
| 7 | AAA24807.1 | outer membrane protein II, partial | | 1 | | 25538 | | 113 | | ***Enterobacter*** *aerogenes* | |
| 8 | ERE53009.1 | 30S ribosomal protein S2, partial | | 1 | | 25562 | | 72 | | *Enterococcus gallinarum* | |
| 9 | BAA32342.1 | ribosomal protein S2 | | 1 | | 31289 | | 72 | | *Pseudomonas aeruginosa* | |

***Sample UR8***

|  | Accession | Protein_description | Σ Peptides | | MW (Da) | | ∑ Score | | Bacteria | |  |
| --- | --- | --- | --- | --- | --- | --- | --- | --- | --- | --- | --- |
| 1 | P24016.1 | RecName: Full=Outer membrane protein A; AltName: Full=Outer membrane protein II | | 3 | | 25648 | | 391 | | *Citrobacter freundii* | |
| 2 | YP_001335792.1 | murein lipoprotein | | 2 | | 8381 | | 290 | | *Klebsiella pneumoniae* | |
| 3 | YP_001452149.1 | outer membrane porin protein C | | 2 | | 40804 | | 242 | | ***Citrobacter koseri*** | |
| 4 | 1006243A | lipoprotein mutant | | 1 | | 8265 | | 204 | | *Escherichia coli* | |
| 5 | YP_001334418.1 | peptidoglycan-associated outer membrane lipoprotein | | 2 | | 18851 | | 165 | | *Klebsiella pneumoniae* | |
| 6 | YP_001337990.1 | 50S ribosomal protein L7/L12 | | 1 | | 12334 | | 163 | | *Klebsiella pneumoniae* | |
| 7 | NP_290617.1 | 50S ribosomal protein L7/L12 | | 1 | | 12288 | | 163 | | *Escherichia coli* | |
| 8 | WP_002438624.1 | 50S ribosomal protein L7/L12 | | 1 | | 12334 | | 151 | | *Escherichia hermannii* | |
| 9 | YP_049963.1 | major outer membrane lipoprotein | | 1 | | 8396 | | 146 | | *Pectobacterium atrosepticum* | |
| 10 | AHF77098.1 | Major outer membrane lipoprotein | | 1 | | 8327 | | 146 | | *Sodalis sp.* | |
| 11 | WP_002438592.1 | outer membrane phosphoporin protein E | | 1 | | 38178 | | 143 | | *Escherichia hermannii* | |
| 12 | WP_023490706.1 | 50S ribosomal protein L7/L12 | | 1 | | 12320 | | 139 | | *Serratia sp.* | |
| 13 | YP_001453952.1 | peptidoglycan-associated outer membrane lipoprotein | | 1 | | 18655 | | 132 | | *Citrobacter koseri* | |
| 14 | YP_001570436.1 | hypothetical protein SARI_01396 | | 1 | | 41232 | | 124 | | *Salmonella enterica* | |
| 15 | NP_456260.1 | heat shock protein | | 1 | | 17671 | | 113 | | *Salmonella enterica* | |
| 16 | YP_005196338.1 | outer membrane pore protein F | | 1 | | / | | 112 | | *Pantoea ananatis* | |
| 17 | AAQ96078.1 | outer membrane protein II | | 1 | | 17424 | | 108 | | *Hafnia alvei* | |
| 18 | YP_001455345.1 | hypothetical protein CKO_03833 | | 1 | | 85023 | | 106 | | *Citrobacter koseri* | |
| 19 | ERE43463.1 | alkyl hydroperoxide reductase subunit C, partial | | 1 | | 20509 | | 91 | | *Enterococcus gallinarum* | |
| 20 | NP_455184.1 | alkyl hydroperoxide reductase c22 protein | | 1 | | 20734 | | 91 | | *Salmonella enterica* | |
| 21 | NP_455305.1 | peptidoglycan-associated lipoprotein | | 1 | | 18853 | | 88 | | *Salmonella enterica* | |
| 22 | ADK27722.1 | outer membrane protein F | | 1 | | / | | 86 | | *Yersinia aldovae* | |
| 23 | P02938.1 | RecName: Full=Major outer membrane lipoprotein; AltName: Full=Murein-lipoprotein; Flags: Precursor | | 1 | | 8234 | | 86 | | *Serratia marcescens* | |
| 24 | YP_003883508.1 | outer membrane protein F | | 1 | | 39421 | | 85 | | *Dickeya dadantii* | |
| 25 | YP_001336542.1 | autonomous glycyl radical cofactor GrcA | | 1 | | 14234 | | 84 | | *Klebsiella pneumoniae* | |
| 26 | P18953.2 | RecName: Full=Autonomous glycyl radical cofactor | | 1 | | 14366 | | 84 | | *Serratia liquefaciens* | |
| 27 | NP_460044.1 | outer membrane protein A | | 1 | | 37492 | | 83 | | *Salmonella enterica* | |
| 28 | YP_001175971.1 | peptidoglycan-associated outer membrane lipoprotein | | 1 | | 18801 | | 79 | | *Enterobacter sp.* | |
| 29 | YP_538610.1 | hypothetical protein UTI89_P011 | | 1 | | 19211 | | 79 | | *Escherichia coli* | |
| 30 | YP_001454596.1 | hypothetical protein CKO_03069 | | 1 | | 9501 | | 76 | | *Citrobacter koseri* | |
| 31 | WP_021542859.1 | hypothetical protein | | 1 | | 9376 | | 75 | | *Escherichia coli* | |
| 32 | YP_004116947.1 | enolase | | 1 | | 45393 | | 74 | | *Pantoea sp.* | |
| 33 | WP_006176733.1 | autonomous glycyl radical cofactor GrcA | | 1 | | 14432 | | 71 | | *Enterobacter* | |
| 34 | NP_457121.1 | autonomous glycyl radical cofactor GrcA | | 1 | | 14335 | | 71 | | *Salmonella enterica* | |
| 35 | YP_001338188.1 | RNA-binding protein Hfq | | 1 | | 10996 | | 69 | | *Klebsiella pneumoniae* | |
| 36 | AAA24175.1 | ORF, partial | | 1 | | 8848 | | 69 | | *Escherichia coli* | |
| 37 | YP_006588965.1 | RNA chaperone Hfq | | 1 | | 11375 | | 69 | | *secondary endosymbiont of Heteropsylla cubana* | |
| 38 | YP_454193.1 | phosphopyruvate hydratase | | 1 | | 45803 | | 68 | | *Sodalis glossinidius* | |
| 39 | EUD03787.1 | outer membrane protein C | | 1 | | / | | 67 | | *Providencia alcalifaciens* | |
| 40 | WP_019677784.1 | 50S ribosomal protein L7/L12 | | 1 | | 12157 | | 67 | | *Rheinheimera perlucida* | |
| 41 | 1LR1_A | Chain A, Solution Structure Of The Oligomerization Domain Of The Bacterial Chromatin-Structuring Protein H-Ns | | 1 | | 7005 | | 67 | | *Escherichia coli* | |
| 42 | WP_024131942.1 | membrane protein | | 1 | | 37713 | | 65 | | *Salmonella enterica* | |
| 43 | YP_001338154.1 | chaperonin GroEL | | 1 | | 57090 | | 64 | | *Klebsiella pneumoniae* | |
| 44 | BAA25225.1 | similar to GroEL protein | | 1 | | 56351 | | 64 | | *Klebsiella pneumoniae* | |
| 45 | ERE50858.1 | outer membrane porin protein C | | 1 | | 40831 | | 62 | | *Enterococcus gallinarum* | |
| 46 | YP_001336754.1 | phosphopyruvate hydratase | | 1 | | 45521 | | 62 | | *Klebsiella pneumoniae subsp. pneumoniae* | |
| 47 | YP_002932251.1 | Gram-negative porin family protein | | 1 | | / | | 62 | | *Edwardsiella ictaluri* | |
| 48 | YP_001336083.1 | outer membrane pore protein | | 1 | | / | | 62 | | *Klebsiella pneumoniae* | |

***Sample UR9***

|  | Accession | Protein_description | Σ Peptides | MW (Da) | ∑ Score | Bacteria |
| --- | --- | --- | --- | --- | --- | --- |
| 1 | YP_002150354.1 | peptidoglycan-associated outer membrane lipoprotein | 4 | 18118 | 650 | ***Proteus mirabilis*** |
| 2 | YP_001334418.1 | peptidoglycan-associated outer membrane lipoprotein | 2 | 18851 | 184 | *Klebsiella pneumoniae* |
| 3 | AAB86977.1 | peptidoglycan associated lipoprotein | 1 | 6732 | 76 | *Allochromatium vinosum* |
| 4 | YP_003147027.1 | peptidoglycan-associated lipoprotein | 1 | 19050 | 76 | *Kangiella koreensis* |
| 5 | WP_017628417.1 | outer membrane porin protein C | 1 | 41496 | 74 | *Proteus mirabilis* |
| 6 | NP_290617.1 | 50S ribosomal protein L7/L12 | 1 | 12288 | 60 | *Escherichia coli* |
| 7 | WP_017842506.1 | peptidoglycan-associated lipoprotein | 1 | / | 60 | *Methylomicrobium buryatense* |
| 8 | YP_048349.1 | 50S ribosomal protein L7/L12 | 1 | 12424 | 60 | *Pectobacterium atrosepticum* |

***Sample UR10***

|  | Accession | Protein_description | Σ Peptides | MW (Da) | ∑ Score | Bacteria |
| --- | --- | --- | --- | --- | --- | --- |
| 1 | YP_002151979.1 | DL-methionine transporter substrate-binding subunit | 2 | 29682 | 295 | ***Proteus mirabilis*** |
| 2 | YP_002150354.1 | peptidoglycan-associated outer membrane lipoprotein | 1 | 18118 | 225 | *Proteus mirabilis* |
| 3 | YP_001334418.1 | peptidoglycan-associated outer membrane lipoprotein | 2 | 18851 | 161 | *Klebsiella pneumoniae* |
| 4 | YP_002150599.1 | outer membrane receptor | 2 | 73933 | 160 | *Proteus mirabilis* |
| 5 | P09461.1 | RecName: Full=Major outer membrane lipoprotein; AltName: Full=Murein-lipoprotein; Flags: Precursor | 1 | 8246 | 119 | *Proteus mirabilis* |
| 6 | NP_455305.1 | peptidoglycan-associated lipoprotein | 1 | 18853 | 104 | *Salmonella enterica* |
| 7 | YP_051614.1 | DL-methionine transporter substrate-binding subunit | 1 | 29463 | 73 | *Pectobacterium atrosepticum* |
| 8 | YP_001333901.1 | DL-methionine transporter substrate-binding subunit | 1 | 29366 | 72 | *Klebsiella pneumoniae* |
| 9 | AAA24507.1 | lipoprotein 28, partial | 1 | 10781 | 72 | *Escherichia coli* |
| 10 | NP_670405.1 | DL-methionine transporter substrate-binding subunit | 1 | / | 72 | *Yersinia pestis* |
| 11 | AAB86977.1 | peptidoglycan associated lipoprotein | 1 | 6732 | 57 | *Allochromatium vinosum* |
| 12 | YP_003147027.1 | peptidoglycan-associated lipoprotein | 1 | / | 56 | *Kangiella koreensis* |

***Sample UR11***

|  | Accession | | Protein_description | Σ Peptides | | MW (Da) | | ∑ Score | | Bacteria | |  |
| --- | --- | --- | --- | --- | --- | --- | --- | --- | --- | --- | --- | --- |
| 1 | | AAA24232.1 | outer membrane protein II, partial | | 1 | | 26114 | | 164 | | *Escherichia fergusonii* | |
| 2 | | NP_287228.1 | acyl carrier protein | | 2 | | 8634 | | 134 | | ***Escherichia coli*** | |
| 3 | | ERE53009.1 | 30S ribosomal protein S2, partial | | 1 | | 25562 | | 82 | | *Enterococcus gallinarum* | |
| 4 | | BAA32342.1 | ribosomal protein S2 | | 1 | | 31289 | | 82 | | *Pseudomonas aeruginosa* | |
| 5 | | YP_001335792.1 | murein lipoprotein | | 1 | | 8381 | | 68 | | *Klebsiella pneumoniae* | |
| 6 | | P02938.1 | RecName: Full=Major outer membrane lipoprotein; AltName: Full=Murein-lipoprotein; Flags: Precursor | | 1 | | 8234 | | 68 | | *Serratia marcescens* | |
| 7 | | 1BXW_A | Chain A, Outer Membrane Protein A (Ompa) Transmembrane Domain | | 1 | | 18864 | | 67 | | *Escherichia coli* | |
| 8 | | YP_001336542.1 | autonomous glycyl radical cofactor GrcA | | 1 | | 14234 | | 65 | | *Klebsiella pneumoniae* | |
| 9 | | P18953.2 | RecName: Full=Autonomous glycyl radical cofactor | | 1 | | 14366 | | 65 | | *Serratia liquefaciens* | |
| 10 | | CAA57795.1 | enolase | | 1 | | 46417 | | 63 | | *Escherichia coli* | |
| 11 | | YP_001334138.1 | adenylate kinase | | 1 | | 23533 | | 61 | | *Klebsiella pneumoniae* | |
| 12 | | BAA14303.1 | adenylate kinase | | 1 | | 12103 | | 61 | | *Escherichia coli* | |

***Sample UR12***

|  | Accession | Protein_description | Σ Peptides | MW (Da) | ∑ Score | Bacteria | |
| --- | --- | --- | --- | --- | --- | --- | --- |
| 1 | YP_002150599.1 | outer membrane receptor | 3 | 73933 | 382 | | ***Proteus mirabilis*** |
| 2 | YP_002150354.1 | peptidoglycan-associated outer membrane lipoprotein | 1 | 18118 | 275 | | *Proteus mirabilis* |
| 3 | YP_002152262.1 | molecular chaperone GroEL | 1 | 57615 | 150 | | *Proteus mirabilis* |
| 4 | NP_927927.1 | molecular chaperone DnaK | 2 | 68818 | 142 | | *Photorhabdus luminescens* |
| 5 | WP_021710396.1 | chaperone protein DnaK | 2 | 69029 | 142 | | *Vibrio azureus* |
| 6 | YP_002150023.1 | phosphoglycerate kinase | 1 | 41197 | 121 | | *Proteus mirabilis* |
| 7 | YP_858397.1 | outer membrane receptor | 1 | 71927 | 72 | | *Aeromonas hydrophila* |
| 8 | YP_002152488.1 | 50S ribosomal protein L10 | 1 | 17640 | 72 | | *Proteus mirabilis* |

***Sample UR13***

|  | Accession | | Protein_description | Σ Peptides | MW (Da) | ∑ Score | Bacteria |
| --- | --- | --- | --- | --- | --- | --- | --- |
| 1 | | YP_004593040.1 | peptidoglycan-associated outer membrane lipoprotein | 1 | 18865 | 160 | ***Enterobacter aerogenes*** |
| 2 | | 1OEL_A | Chain A, Conformational Variability In The Refined Structure Of The Chaperonin | 1 | 57031 | 157 | *Escherichia coli* |
| 3 | | YP_001337379.1 | elongation factor G | 1 | 77494 | 98 | *Klebsiella pneumoniae* |
| 4 | | NP_289887.1 | elongation factor G | 1 | 77532 | 98 | *Escherichia coli* |
| 5 | | YP_001334507.1 | DNA starvation/stationary phase protection protein Dps | 1 | 18697 | 89 | *Klebsiella pneumoniae* |
| 6 | | AAB49426.1 | PexB, partial | 1 | 5584 | 89 | *Salmonella enterica* |
| 7 | | YP_001334652.1 | outer membrane protein A | 1 | 38021 | 85 | *Klebsiella pneumoniae* |
| 8 | | P09146.1 | RecName: Full=Outer membrane protein A; Flags: Precursor | 1 | 37552 | 85 | *Klebsiella aerogenes* |
| 9 | | YP_004393584.1 | major outer membrane protein OmpAII | 1 | 36565 | 83 | *Aeromonas veronii* |
| 10 | | WP_021015441.1 | Ferritin Dps family protein | 1 | / | 74 | *Serratia sp.* |

***Sample UR14***

|  | Accession | Protein_description | Σ Peptides | MW (Da) | ∑ Score | Bacteria |
| --- | --- | --- | --- | --- | --- | --- |
| 1 | YP_001335792.1 | murein lipoprotein | 1 | 8381 | 188 | *Klebsiella pneumoniae* |
| 2 | P02938.1 | RecName: Full=Major outer membrane lipoprotein; AltName: Full=Murein-lipoprotein; Flags: Precursor | 1 | 8234 | 188 | *Serratia marcescens* |
| 3 | AAA24807.1 | outer membrane protein II, partial | 2 | 25538 | 140 | ***Enterobacter*** *aerogenes* |
| 4 | YP_049963.1 | major outer membrane lipoprotein | 1 | 8396 | 139 | *Pectobacterium atrosepticum* |
| 5 | AHF77098.1 | Major outer membrane lipoprotein | 1 | 8327 | 139 | *Sodalis sp.* |
| 6 | P24016.1 | RecName: Full=Outer membrane protein A; AltName: Full=Outer membrane protein II | 1 | 25648 | 77 | *Citrobacter freundii* |

***Sample UR15***

|  | Accession | | Protein_description | Σ Peptides | MW (Da) | ∑ Score | Bacteria |
| --- | --- | --- | --- | --- | --- | --- | --- |
| 1 | | NP_814526.1 | cold shock domain-contain protein | 1 | 7109 | 94 | ***Enterococcus faecalis*** |
| 2 | | YP_001335792.1 | murein lipoprotein | 1 | 8381 | 90 | *Klebsiella pneumoniae* |
| 3 | | P02938.1 | RecName: Full=Major outer membrane lipoprotein; AltName: Full=Murein-lipoprotein; Flags: Precursor | 1 | 8234 | 90 | *Serratia marcescens* |
| 4 | | WP_005297706.1 | glyceraldehyde-3-phosphate dehydrogenase | 1 | 40057 | 64 | *Photobacterium damselae* |
| 5 | | WP_009972002.1 | hypothetical protein | 1 | 4391 | 59 | *Burkholderia pseudomallei* |
| 6 | | YP_002602369.1 | protein FeoB1 | 1 | / | 55 | *Desulfobacterium autotrophicum* |
| 7 | | ETT00120.1 | TcdA/TcdB pore forming domain protein | 1 | / | 55 | *Providencia alcalifaciens* |
| 8 | | NP_643074.1 | PmbA protein | 1 | / | 54 | *Xanthomonas axonopodis* |
| 9 | | WP_020491921.1 | UDP-N-acetylenolpyruvoylglucosamine reductase | 1 | 31795 | 53 | *Dehalobacter sp.* |

***Sample UR16***

|  | Accession | | Protein_description | Σ Peptides | MW (Da) | ∑ Score | Bacteria |
| --- | --- | --- | --- | --- | --- | --- | --- |
| 1 | | YP_049963.1 | major outer membrane lipoprotein | 1 | 8396 | 212 | *Pectobacterium atrosepticum* |
| 2 | | AHF77098.1 | Major outer membrane lipoprotein | 2 | 8327 | 212 | *Sodalis sp.* |
| 3 | | YP_001335792.1 | murein lipoprotein | 1 | 8381 | 161 | ***Klebsiella pneumoniae*** |
| 4 | | P02938.1 | RecName: Full=Major outer membrane lipoprotein; AltName: Full=Murein-lipoprotein; Flags: Precursor | 1 | 8234 | 161 | *Serratia marcescens* |
| 5 | | YP_001463566.1 | outer membrane porin protein C | 1 | 40070 | 102 | ***Escherichia coli*** |
| 6 | | ABS84845.1 | translation elongation factor Tu | 1 | 21970 | 85 | *Bacillus subtilis* |
| 7 | | NP_299905.1 | elongation factor Tu | 1 | 42849 | 85 | *Xylella fastidiosa* |
| 8 | | 0803214A | elongation factor Tu 59-263 | 1 | 22630 | 85 | *Escherichia coli* |
| 9 | | NP_667815.1 | elongation factor Tu | 1 | / | 84 | *Yersinia pestis* |
| 10 | | AAQ96088.1 | outer membrane protein II | 1 | 17373 | 62 | *Klebsiella pneumoniae* |
| 11 | | YP_003929643.1 | hypothetical protein Pvag_pPag10133 | 1 | / | 61 | *Pantoea vagans* |
| 12 | | YP_001337372.1 | 50S ribosomal protein L4 | 1 | 22100 | 59 | *Klebsiella pneumoniae* |
| 13 | | NP_289880.1 | 50S ribosomal protein L4 | 1 | 22073 | 59 | *Escherichia coli* |

**Supplementary table 5: The comparative view of urine culture, proteomics and genomic results.**

| **N.o.** | **Urine culture identification** | **MALDI TOF/TOF IDENTIFICATION** | **16S rRNA sequencing identification** |
| --- | --- | --- | --- |
| UR1 | *Klebsiella pneumoniae* | *Klebsiella pneumoniae* | *100% Enterobacteriaceae* |
| UR2 | *Klebsiella oxytoca* | *Klebsiella pneumoniae* | *97% Enterobacteriaceae* |
| UR3 | *Klebsiella pneumoniae* | *Klebsiella pneumoniae* | *90% Enterobacteriaceae, 5.1% Granulicatella, 1,1% Anaerococcus* |
| UR4 | *Proteus mirabilis* | *Proteus mirabilis* | *97.7% Proteus, 1% Enterobacteriaceae* |
| UR5 | *Enterococcus faecalis* | *Enterococcus faecalis* | *21,8% Pseudomonas; 13,7% Propionibacterium acnes; 11% Lactobacillus helvetisus; 8,1% Adhaeribacter; 8% Acinetobacter; 5,9% Staphylococcus; 4,9% Stenotrophomonas; 3,8% Hydrogenophaga; 3,6% Erysipelotrichaceae; 3,1% Corynebacterium; 3,1% Cellulomonas; 2,3% Aerococcus; 2% Acidovorax; 1,9% Lachnospiraceae; 1,6% Sphingobium* |
| UR6 | *Enterococcus faecalis* | *Enterococcus faecalis* | *51,4% Enterococcus, 46,5% Enterococcaceae* |
| UR7 | *Enterobacter cloaceae* | *Citrobacter freundii* | *98% Enterobacter; 0,9% Proteus* |
| UR8 | *Citrobacter koseri* | *Citrobacter freundii* | *57,5% Citrobacter koseri; 4,5% Bacteroides; 3,7% Dysgonomonas; 2,7% Bacteroides; 2,6% Rikenellaceae; 2,3% Parabacteroides; 2% Desulfovibrionaceae; 2% Lachnospiraceae; 2% Ruminococcaceae; 1,9% Enterobacteriaceae; 1,3% Ruminococcus; 1,3% Erysipelotrichaceae; 1,2% Enterococcus; 1,1% Clostridiales* |
| UR9 | *Proteus mirabilis* | *Proteus mirabilis* | *96.7% Proteus; 2,4% Enterobacteriaceae; 1,2% Prevotella* |
| UR10 | *Proteus mirabilis* | *Proteus mirabilis* | *97% Proteus; 1,3% Enterobacteriaceae* |
| UR11 | *Escherichia coli; Proteus mirabilis* | *Escherichia fergusonii* | *93% Enterobacteriaceae; 3,5% Proteus* |
| UR12 | *Proteus mirabilis* | *Proteus mirabilis* | *99,2% Proteus* |
| UR13 | *Enterobacter aerogenes* | *Enterobacter aerogenes* | *75,4% Enterobacteriaceae; 14% Lactobacillus delbrueckii; 4,2% Kluyvera; 4% Enterobacter; 1% Lactobacillus helveticus* |
| UR14 | *Enterobacter cloacae* | *Klebsiella pneumoniae* | *95,4% Enterobacteriaceae; 1,2% Clostridium perfringens; 1% Bifidobacterium pseudolongum* |
| UR15 | *Enterobacter cloacae; Enterococcus faecalis;  E coli;  Proteus mirabilis* | *Enterococcus faecalis* | *86,9% Proteus; 7,2% Enterobacteriaceae; 2,2% Enterobacter; 1% Rhodospirillaceae* |
| UR16 | *Escherichia coli;  Klebsiella pneumoniae* | *Pectobacterium atrosepticum* | *91,2% Enterobacteriaceae; 8,6% Klebsiella* |

**Supplementary table 6: Identified human proteins ranked by MASCOT score for each urine sample.**

***Sample UR1***

|  | Accession | Protein_description | Score | ∑ Peptide |
| --- | --- | --- | --- | --- |
| 1 | BAG59381.1 | unnamed protein product | 140 | 1 |
| 2 | CAA34982.1 | alpha-1 antitrypsin | 99 | 1 |
| 3 | CAA26677.1 | alpha-1-antitrypsin (aa 268-394) | 70 | 1 |
| 4 | NP_000691.1 | annexin A1 | 67 | 1 |
| 5 | 740525A | lipoprotein Gln I | 66 | 1 |
| 6 | 2A01_A | Chain A, Crystal Structure Of Lipid-Free Human Apolipoprotein A-I | 66 | 1 |
| 7 | CAA37116.1 | unnamed protein product | 55 | 1 |

***Sample UR2***

|  | Accession | Protein_description | Score | ∑ Peptide |
| --- | --- | --- | --- | --- |
| 1 | CAA23759.1 | unnamed protein product | 325, 200, 105 | 3 |
| 2 | 2YRS_B | Chain B, Human Hemoglobin D Los Angeles: Crystal Structure | 325 | 1 |
| 3 | AAN84548.1 | beta globin chain variant | 283 | 1 |
| 4 | 1NQP_B | Chain B, Crystal Structure Of Human Hemoglobin E At 1.73 A Resolution | 239 | 1 |
| 5 | AAY46275.1 | beta globin chain | 214 | 1 |
| 6 | AAY51976.1 | hemoglobin beta chain | 200 | 1 |
| 7 | AAA36799.1 | uromodulin | 170, 51 | 2 |
| 8 | CAA23749.1 | alpha globin | 152 | 1 |
| 9 | AAA51747.1 | proapolipoprotein, partial | 105, 76 | 2 |
| 10 | 740525A | lipoprotein Gln I | 100 | 1 |
| 11 | 2A01_A | Chain A, Crystal Structure Of Lipid-Free Human Apolipoprotein A-I | 100 | 1 |
| 12 | ACF16774.1 | beta globin | 85 | 1 |
| 13 | AAA35597.1 | beta-globin | 64 | 1 |
| 14 | 1Y5K_B | Chain B, T-to-t(high) Quaternary Transitions In Human Hemoglobin: Betad99a Deoxy Low-salt (10 Test Sets) | 64 | 1 |

***Sample UR3***

|  | Accession | Protein_description | Score | ∑ Peptide |
| --- | --- | --- | --- | --- |
| 1 | AAA36799.1 | uromodulin | 121, 91 | 2 |
| 2 | NP_002956.1 | protein S100-A9 | 101, 86 | 2 |
| 3 | AAB50880.1 | anitubulin IgG1 kappa VL chain {N-terminal} [human, serum, immunocytic sarcom patient PER isolate, Peptide Partial, 219 aa] | 89 | 1 |
| 4 | 1COH_A | Chain A, Structure Of Haemoglobin In The Deoxy Quaternary State With Ligand Bound At The Alpha Haems | 82 | 1 |
| 5 | CAA23759.1 | unnamed protein product | 75 | 1 |
| 6 | 3KR3_L | Chain L, Crystal Structure Of Igf-Ii Antibody Complex | 70 | 1 |
| 7 | BAG59381.1 | unnamed protein product | 65 | 1 |
| 8 | AAA51747.1 | proapolipoprotein, partial | 55 | 1 |
| 9 | 740525A | lipoprotein Gln I | 54 | 1 |

***Sample UR4***

|  | Accession | Protein_description | Score | ∑ Peptide |
| --- | --- | --- | --- | --- |
| 1 | NP_002956.1 | protein S100-A9 | 89 | 1 |
| 2 | CAA68390.1 | unnamed protein product | 61 | 1 |
| 3 | AAH13767.1 | VASN protein | 58 | 1 |

***Sample UR5***

|  | Accession | Protein_description | Score | ∑ Peptide |
| --- | --- | --- | --- | --- |
| 1 | AAA36799.1 | uromodulin | 148, 74 | 2 |
| 2 | CAA23749.1 | alpha globin | 52 | 1 |

***Sample UR6***

|  | Accession | Protein_description | Score | ∑ Peptide |
| --- | --- | --- | --- | --- |
| 1 | AAA51747.1 | proapolipoprotein, partial | 475, 344, 195 | 3 |
| 2 | 740525A | lipoprotein Gln I | 354, 234, 195, 69 | 4 |
| 3 | AAB59495.1 | alpha-1-antitrypsin | 132, 59 | 2 |
| 4 | 1OR3_A | Chain A, Apolipoprotein E3 (Apoe3), Trigonal Truncation Mutant 165 | 131 | 1 |
| 5 | AAA51547.1 | alpha-1-antitrypsin precursor | 104 | 1 |
| 6 | AAB59372.1 | apolipoprotein C-III | 91 | 1 |
| 7 | AAB59518.1 | apolipoprotein E | 71, 60 | 2 |
| 8 | CAA23749.1 | alpha globin | 65 | 1 |
| 9 | 1506383A | apolipoprotein E mutant E3K | 52 | 1 |

***Sample UR7***

|  | Accession | Protein_description | Score | ∑ Peptide |
| --- | --- | --- | --- | --- |
| 1 | CAA23749.1 | alpha globin | 95 | 1 |
| 2 | NP_002956.1 | protein S100-A9 | 91 | 1 |
| 3 | 2CMR_L | Chain L, Crystal Structure Of The Hiv-1 Neutralizing Antibody D5 Fab Bound To The Gp41 Inner-Core Mimetic 5-Helix | 84 | 1 |
| 4 | CAA23759.1 | unnamed protein product | 70 | 1 |
| 5 | 3KR3_L | Chain L, Crystal Structure Of Igf-Ii Antibody Complex | 66 | 1 |
| 6 | 4JFY_A | Chain A, Apo Structure Of Phosphotyrosine (pyab) Scaffold | 77 | 1 |

***Sample UR8***

|  | Accession | Protein_description | Score | ∑ Peptide |
| --- | --- | --- | --- | --- |
| 1 | AAM13690.1 | delta 2-isopentenyl pyrophosphate transferase-like protein | 69 | 1 |

***Sample UR9***

|  | Accession | Protein_description | Score | ∑ Peptide |
| --- | --- | --- | --- | --- |
| 1 | AAA51747.1 | proapolipoprotein, partial | 212, 87 | 2 |
| 2 | CAA34982.1 | alpha-1 antitrypsin | 68 | 1 |
| 3 | AAA36799.1 | uromodulin | 50 | 1 |

***Sample UR10***

|  | Accession | Protein_description | Score | ∑ Peptide |
| --- | --- | --- | --- | --- |
| 1 | AAA36799.1 | uromodulin | 119 | 1 |
| 2 | A37927 | Ig kappa chain C region (allotype Inv(1,2)) - human (fragment) | 114 | 1 |
| 3 | 3KR3_L | Chain L, Crystal Structure Of Igf-Ii Antibody Complex | 114 | 1 |
| 4 | NP_002956.1 | protein S100-A9 | 86 | 1 |
| 5 | CAA33438.1 | unnamed protein product | 61 | 1 |
| 6 | CAA40416.1 | histone H2A.2 | 57 | 1 |
| 7 | 740525A | lipoprotein Gln I | 56 | 1 |
| 8 | CAA68390.1 | unnamed protein product | 44 | 1 |
| 9 | CAA37116.1 | unnamed protein product | 42 | 1 |

***Sample UR11***

|  | Accession | Protein_description | Score | ∑ Peptide |
| --- | --- | --- | --- | --- |
| 1 | AAA36799.1 | uromodulin | 119 | 1 |
| 2 | AAF00488.1 | hemoglobin beta subunit variant | 43 | 1 |

***Sample UR12***

|  | Accession | Protein_description | Score | ∑ Peptide |
| --- | --- | --- | --- | --- |
| 1 | 1AU8_A | Chain A, Human Cathepsin G | 71 | 1 |
| 2 | AAB57795.1 | lactoferrin | 53 | 1 |
| 3 | NP_001148.1 | annexin A11 isoform 1 | 41 | 1 |

***Sample UR13***

|  | Accession | Protein_description | Score | ∑ Peptide |
| --- | --- | --- | --- | --- |
| 1 | AAA36799.1 | uromodulin | 149 | 1 |
| 2 | NP_002956.1 | protein S100-A9 | 85 | 1 |

***Sample UR14***

|  | Accession | Protein_description | Score | ∑ Peptide |
| --- | --- | --- | --- | --- |
| 1 | CAA23759.1 | unnamed protein product | 104, 69 | 2 |
| 2 | CAA23749.1 | alpha globin | 99, 77 | 2 |

***Sample UR15***

|  | Accession | Protein_description | Score | ∑ Peptide |
| --- | --- | --- | --- | --- |
| 1 | CAA23759.1 | unnamed protein product | 244, 138, 119 | 3 |
| 2 | AAF00488.1 | hemoglobin beta subunit variant | 220 | 1 |
| 3 | BAA33580.1 | anti-HBsAg immunoglobulin Fab kappa chain | 123 | 1 |
| 4 | CAA23749.1 | alpha globin | 112 | 1 |
| 5 | AAY46275.1 | beta globin chain | 104 | 1 |
| 6 | AAA35597.1 | beta-globin | 104, 71 | 2 |
| 7 | 3KR3_L | Chain L, Crystal Structure Of Igf-Ii Antibody Complex | 103 | 1 |
| 8 | AAA36799.1 | uromodulin | 66 | 1 |
| 9 | H7BZ55.3 | RecName: Full=Putative ciliary rootlet coiled-coil protein-like 3 protein | 39 | 1 |

***Sample UR16***

|  | Accession | Protein_description | Score | ∑ Peptide |
| --- | --- | --- | --- | --- |
| 1 | protein S100-A9 | NP_002956.1 | 97, 68 | 2 |
